# Direct round window stimulation with the Med-El Vibrant Soundbridge: 5 years of experience using a technique without interposed fascia

**DOI:** 10.1007/s00405-013-2432-1

**Published:** 2013-03-20

**Authors:** Henryk Skarzynski, Lukasz Olszewski, Piotr H. Skarzynski, Artur Lorens, Anna Piotrowska, Marek Porowski, Maciej Mrowka, Adam Pilka

**Affiliations:** 1 Institute of Physiology and Pathology of Hearing, ul Mochnackiego 10, 02-042 Warsaw, Poland; 2 World Hearing Center, ul Mokra 17,Kajetany, 05-830 Nadarzyn, Poland; 3 Institute of Sensory Organs, ul Mokra 1, Kajetany, 05-830 Nadarzyn, Poland

**Keywords:** Hearing loss, Middle ear implant, Vibrant Soundbridge, Round window implants, Partial deafness treatment, Radical cavity, Cochlea

## Abstract

The objective of this study was to present 5 years of surgical experience, and the extended results of hearing preservation (based on 3-year follow-up), with the Med-El Vibrant Soundbridge (VSB) in which the floating mass transducer (FMT) is placed directly against the round window membrane, and the fascia is used only as covering tissue to keep it in position. A retrospective survey of surgical and audiological data was conducted to evaluate the performance and stability of patient hearing, with audiometric measurements performed over fixed time intervals up to 36 months. 21 patients, aged 19–62 years (mean 48.4), with mixed or conductive, bilateral or unilateral hearing loss were included in this study. Surgical intervention involved monaural implantation of the Med-El VSB between 2006 and 2009. The results were assessed using pure tone audiometry. In 5 years of experience with the technique, no significant complications or device extrusion were observed except for two revision surgeries requiring FMT repositioning. In the 3-year follow-up, we observed stable hearing in the implanted ear. It is concluded that direct round window stimulation without interposed fascia is an alternative for patients with hearing impairment caused by chronic otitis media and/or lack of ossicles, especially after modified radical mastoidectomy. It allows good results in a selected group of patients, although further observation on a larger population is needed to confirm long-term validity and effectiveness.

## Introduction

The Med-El Vibrant Soundbridge (VSB) was introduced in Europe in the late 1990s as a middle ear implantable hearing device that could compensate for sensorineural hearing loss. However, it took some time, until 2006, for it to be used for conductive hearing loss treatment [2]. The new method was based on the direct stimulation of the round window (RW) membrane with a standard floating mass transducer (FMT), without a titanium clip and with a fascia interposed between the FMT and the RW membrane. Wider indications for using this type of device led to the development of alternative surgical techniques, such as direct stimulation without interposed tissue.

Previous reports on both methods of round window membrane stimulation presented by Nakajima et al. [3] and Arnold et al. [1] have indicated better results in energy transfer to the cochlea in the direct mode with interposed tissue. However as Pennings et al. [4] justifiably remark, the authors of previous studies analyzed the results of direct placement of the FMT against the RW without a covering fascia, and in some cases, the RW niche had not been drilled off. Pennings and colleagues contend that these factors could affect the results.

Pennings et al. demonstrated that when using an additional covering fascia there was no large difference between both methods in energy transferred to the cochlea. The authors also drew attention to possible future complications that could involve degradation due to scarring of the fascia between the FMT and the RW membrane. However, the current state of knowledge does not allow firm conclusions to be drawn, because a longer period of observation is required. There is little doubt that success in proper positioning of the FMT against the RW membrane is related to the surgeon’s experience.

The results for improving speech intelligibility and quality of hearing have already been well documented in the literature, particularly for sensorineural hearing losses, when the FMT is positioned on the long process of the incus [5]. In comparison, the results of patients with conductive and mixed hearing loss are few. However, they do allow us to conclude that the benefit from using the Med-El VSB system is comparable to other prostheses.

**Fig. 1:**
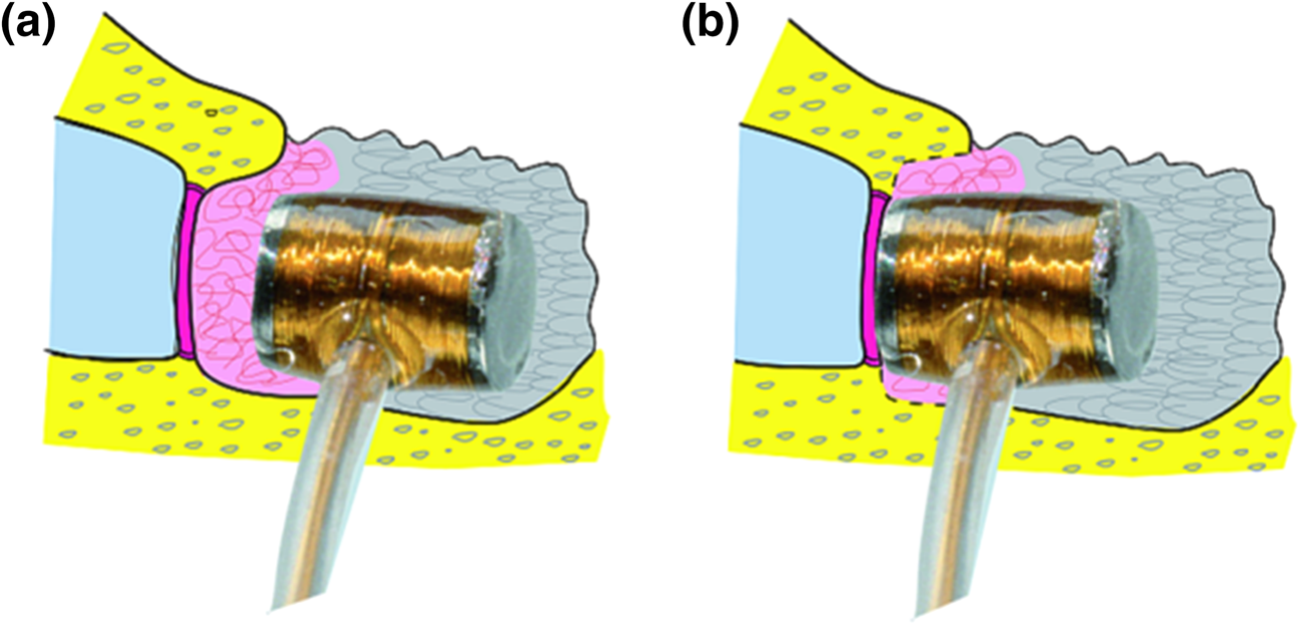
Placement of the FMT against the RW membrane: **a** direct stimulation of RW with interposed tissue and **b** direct stimulation of RW without interposed tissue

The aim of this study was to present 5 years of surgical experience, and extended results of hearing preservation, during 3-year follow-up of patients implanted with the Med-El VSB device in which there was direct placement of the FMT against the RW using a covering fascia over the FMT, but with nothing placed between the FMT and the RW membrane (without interposed tissue) (Fig. 1).

## Materials and methods

Exactly 21 patients, 16 women and 5 men, aged 19–62 years (mean 48.4) with conductive or mixed hearing loss were included in this study; they were implanted at the Institute of Physiology and Pathology of Hearing in Warsaw between 2006 and 2009. Of the 21, 6 were diagnosed with unilateral hearing loss. Demographic data are presented in Table 1.

**Table 1:** Demographic and clinical data

Subject	Implanted ear	Sex	Age at surgery	Hearing loss		Diagnosis
				Implanted ear	Opposite ear	
1	L	F	48	MHL	MHL	CSOM
2	L	M	60	MHL	MHL	CSOM
3	L	M	56	MHL	CHL	CSOM
4	R	F	53	CHL	NH	CSOM
5	R	F	60	CHL	MHL	CSOM
6	L	F	55	MHL	MHL	CSOM
7	L	F	56	MHL	NH	CSOM
8	R	M	43	MHL	NH	CSOM
9	R	F	56	CHL	NH	CSOM
10	L	F	58	MHL	NH	CSOM
11	L	M	33	MHL	MHL	CSOM
12	L	F	27	CHL	CHL	CSOM
13	R	M	19	CHL	NH	CSOM
14	L	F	38	CHL	CHL	CSOM
15	L	F	52	CHL	CHL	CSOM
16	R	F	53	MHL	MHL	CSOM
17	L	F	57	MHL	MHL	CSOM
18	R	F	49	MHL	MHL	CSOM
19	R	F	49	CHL	CHL	CSOM
20	L	F	32	CHL	CHL	CSOM
21	R	F	62	CHL	CHL	CSOM

*MHL* mixed hearing loss, *CHL* conductive hearing loss, *NH* normal hearing, *CSOM* chronic suppurative otitis media

All patients were unilaterally implanted with the Med-El VSB device with the FMT placed directly in contact with the RW membrane (without interposed tissue). Only a few patients used hearing aids prior to the operation since in the majority of cases it was contraindicated due to recurring ear effusions. For at least 1 year before implantation, there was no significant progress in the patients’ hearing losses. None of them complained of tinnitus and/or vertigo before the surgery. Patients had undergone surgical treatment—radical or radical modified mastoidectomy in one or both ears—before they were selected for implantation (Table 2). They all met the audiological criteria for VSB specified by the manufacturer of the device.

**Table 2:** Surgical history of patients

Subject	Type of previous surgery techniques	No. of surgeries before VSB in implanted ear	Main findings during FMT implantation	Postoperative adverse effects
1	Modified radical mastoidectomy	2	None	None
2	Radical mastoidectomy	1	None	None
3	Modified radical mastoidectomy	3	adhesions near RW area	None
4	Modified radical mastoidectomy	2	facial nerve exposure	None
5	Modified radical mastoidectomy	3	None	None
6	radical mastoidectomy	1	None	tinnitus
7	Modified radical mastoidectomy	3	adhesions near RW area	None
8	Modified radical mastoidectomy	3	None	None
9	Modified radical mastoidectomy	6	None	None
10	Modified radical mastoidectomy	5	adhesions near RW area	None
11	Modified radical mastoidectomy	5	None	FMT dislocation
12	Modified radical mastoidectomy	7	adhesions near RW area	FMT dislocation
13	radical mastoidectomy	5	None	None
14	Modified radical mastoidectomy	3	adhesions near RW area	None
15	Modified radical mastoidectomy	4	None	tinnitus, vertigo
16	radical mastoidectomy	3	None	tinnitus
17	Modified radical mastoidectomy	2	None	None
18	modified radical mastoidectomy	4	None	None
19	Modified radical mastoidectomy	2	None	None
20	Modified radical mastoidectomy	3	adhesions near RW area	tinnitus
21	Modified radical mastoidectomy	2	adhesions near RW area	None

The patients were all assessed preoperatively and postoperatively over fixed time intervals to obtain hearing thresholds for both AC and BC at standard frequencies. The intervals were 0–3 months pre-op (pre); 0–1 month (post-interval I); 2–3 months (post-interval II); 4–6 months (post-interval III); 7–12 months (post-interval IV); 13–24 months (post-interval V); and 25–36 months (post-interval VI). Processors were fitted with a DSL I/O formula based on the manufacturer’s recommendations. BC thresholds were used as input data to the calculation of electroacoustic parameters. In cases where there were no acceptable calculated DSL targets, further adjustment was performed on the basis of the subjective preferences of the patient.

## Surgery

Exactly 21 patients were selected for VSB implantation with the FMT placed directly in contact with the RW (without interposed tissue); surgery was done at the International Center of Hearing and Speech of the Institute of Physiology and Pathology of Hearing, Warsaw.

In all patients, an approach through the external ear canal was performed to assess the condition of the middle ear and to visualize the RW niche. In seven patients, the RW niche was occluded by adhesions as a result of chronic otitis and sometimes multiple previous surgeries; in these cases, the adhesions and scar tissue were carefully removed without damaging the RW membrane until the RW could be properly visualized. In all cases, to properly visualize the RW, the bony lip was removed with a small-diameter diamond burr. The drilling was done intermittently at slow speed and with irrigation, always avoiding contact with the RW membrane, because direct physical contact from a working drill can cause sensorineural hearing loss. If possible, only the internal part of the bony lip was removed, leaving the bony roof to form a kind of a well for the FMT. If this was not possible, the whole superior lip was completely drilled away to visualize the RW membrane. At this stage, the template was placed onto the RW, and a postauricular groove was cut. The epidermis lining the postoperative cavity was then carefully detached from the bony bed of the temporal bone to receive the internal part of the implant. A second placement check with the template was done before the FMT was placed in position. We recommend that the FMT is fixed from behind and inferiorly using a piece of fascia and fibrin glue. Next, two or three layers of larger pieces of fascia are used as an additional cover of FMT and the RW, preventing the transducer from moving. After fixing all layers to the surrounding bony surfaces with glue, everything is covered with the earlier detached parts of the epidermis taken from the external ear canal or the postoperative cavity.

The final surgical steps involve placement of the internal capsule of the implant in the earlier prepared bony bed. For the next 7–8 days, the FMT and surrounding structures are covered by a foil dressing.

## Results

**Fig. 2:**
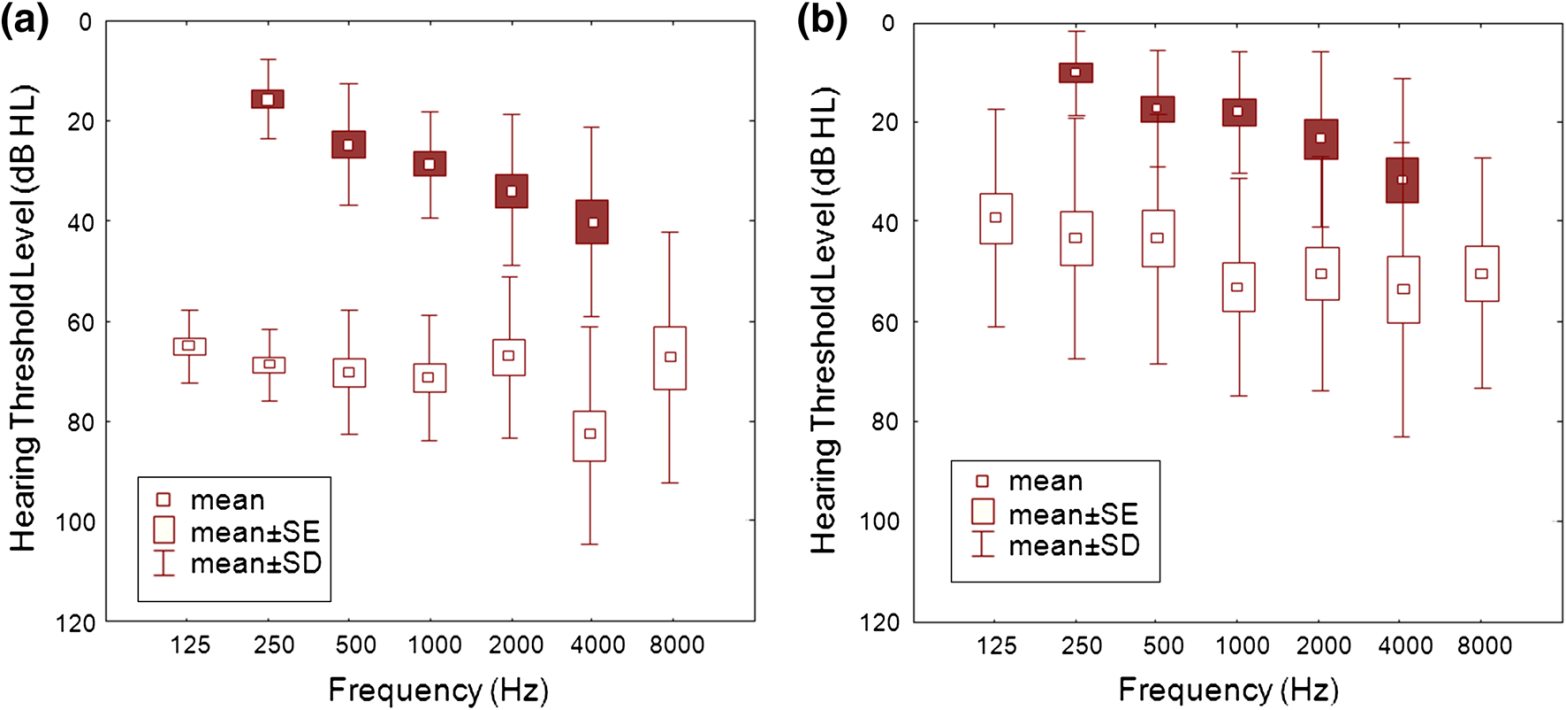
Mean preoperative AC thresholds (*white squares*) and BC thresholds (*black squares*) in the ear chosen for implantation (**a**) and in the opposite ear (**b**)

The results of AC and BC hearing in the ear selected for surgery and in the opposite ear before implantation of the FMT are shown in Fig. 2. On the basis of BC and AC thresholds, it can be seen that 11 patients had mixed hearing loss and 10 conductive hearing loss in the ear selected for surgery. In the opposite ear, 6 patients had normal hearing, 8 mixed, and 7 conductive hearing loss. All statistical analyses were done using the Wilcoxon–Cox rank-sum test.

**Fig. 3:**
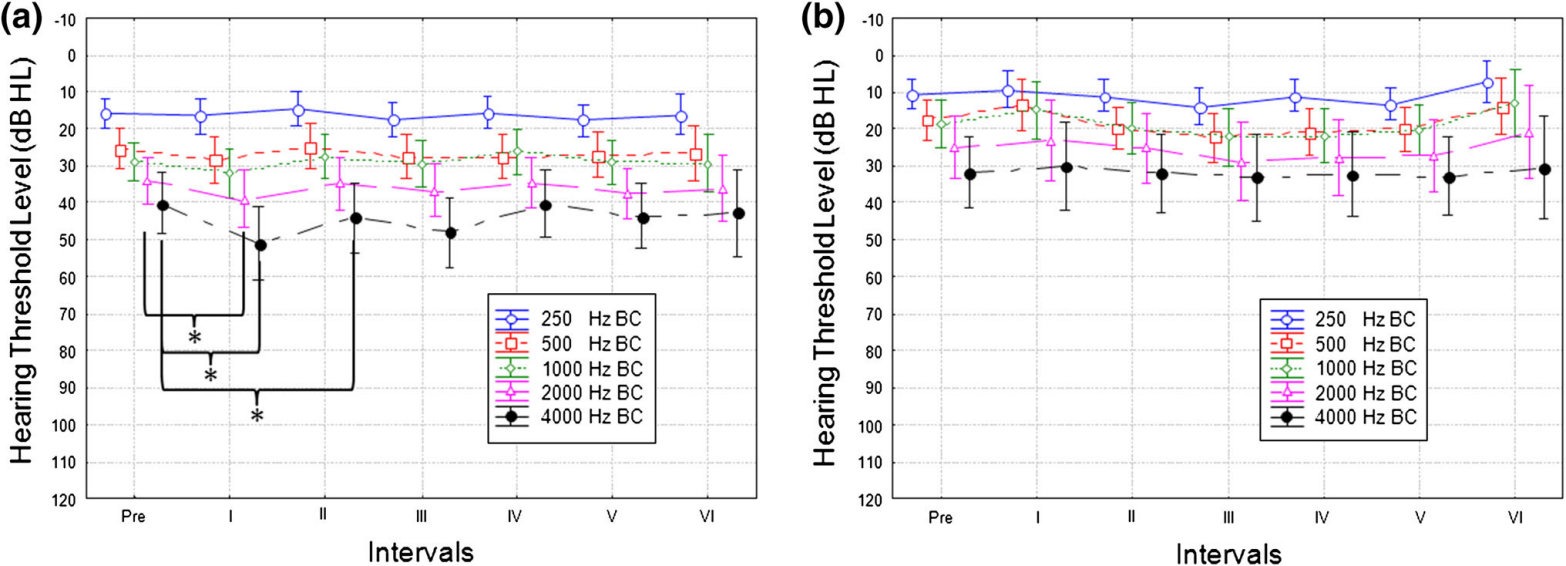
Mean pre and postoperative BC thresholds in the operated ear (**a**) and opposite ear (**b**) over fixed time intervals. The *bars* show 0.95 confidence interval

**Fig. 4:**
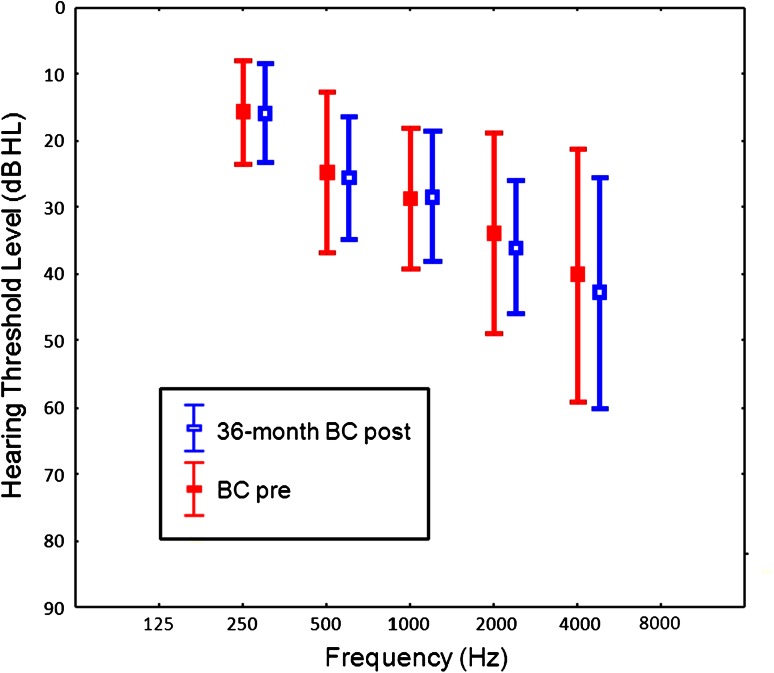
Mean preoperative and 36-month postoperative BC thresholds. The *bars* show 1 standard deviation

Over the fixed time intervals, postoperative BC thresholds in the implanted ear were stable for most frequencies. However, statistical differences between BC thresholds were observed for interval I vs. pre for 2,000 Hz (*p* = 0.043) and 4,000 Hz (*p* = 0.015), and between interval II vs. pre for 4,000 Hz (*p* = 0.03). Comparison of hearing for BC thresholds before and 36 months after direct placement of FMT against the RW showed no statistically significant differences for all tested frequencies (Fig. 3). In the opposite ear, BC thresholds were stable over the whole frequency range during the 36-month follow-up period, confirming threshold stability (Fig. 4).

**Fig. 5:**
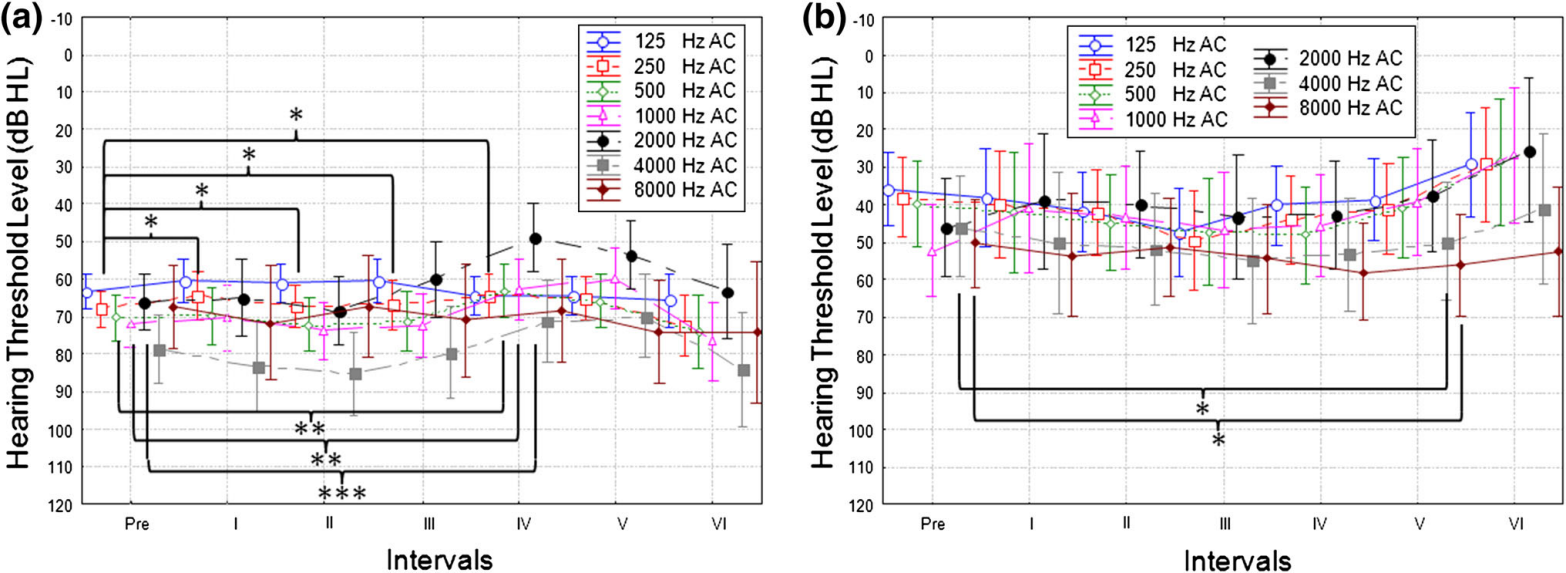
Mean pre and postoperative AC thresholds in the operated ear (**a**) and opposite ear (**b**) over fixed time intervals. The *bars* show 0.95 confidence interval

In terms of AC thresholds in the implanted ear, the analysis shows significant differences between hearing thresholds at 250 Hz before and after implantation up to 1 year after FMT implantation and over each interval (interval I, *p* = 0.04; interval II, *p* = 0.04; interval III, *p* = 0.03; interval IV, *p* = 0.05). Statistically significant changes were also observed between pre and interval IV thresholds at 500, 1,000, and 2,000 Hz (*p* = 0.006, 0.002, 0.001, respectively). In the non-operated ear, we noted statistically significant changes between pre and interval V thresholds at frequencies of 4,000 and 8,000 Hz (*p* = 0.03 and 0.05, respectively) Fig. 5.

## Discussion and conclusions

After 5 years of experience with the use of direct placement of the FMT against the RW using the technique suggested by Skarzynski, the data show that this method of treatment could be an alternative to the commonly used direct approach with fascia between FMT and the RW. The absence of an interposed fascia appears to be an advantage of this method, in terms of assuring better coupling between the FMT and the RW through averting later occurrence of scars and adhesions.

The long-term assessment of hearing preservation after implantation of the Med-El Vibrant Soundbridge device with direct connection of the FMT with the RW membrane was also evaluated in this study.

A 3-year follow-up of the presented group of patients did not show any significant changes in BC thresholds in the operated ear, and so there was no need to significantly change the processor parameters during this time. If any such change is required, it suggests deterioration and instability of the coupling between the RW membrane and the FMT. These results confirm the safety of the surgical method used and its atraumaticity for hearing.

In all cases, the decision to implant was dictated by medical and audiological contraindications to the use of conventional hearing aids. However, it should be emphasized that for this type of hearing disorder other alternative treatments using implantable devices are also available.

The most frequently reported adverse effect after surgery was tinnitus (4 out of 21, 19 %). However, in all cases, it disappeared within 3 months of surgery. One patient reported periodic vertigo. In two cases, when there was a sudden deterioration in hearing (while the VSB device was still working properly), it was necessary to perform revision surgery to reposition the FMT. After that procedure, hearing with the VSB device was restored. The incidence of adverse effects did not differ from other similar reports.

AC threshold shifts for 250 Hz in the operated ear during the first year after surgery suggest that it might be due to the healing process in the middle ear after FMT implantation. After 12 months, AC threshold shifts were no longer significant.

The nature of other significant AC threshold changes is difficult to interpret, and the authors believe that they might be related to the character of conductive and mixed hearing loss, which usually shows periodic fluctuations. The changes may also reflect some other unidentified phenomena occurring in the middle ear after implantation.

Significant changes in BC thresholds observed within the first two intervals for 2,000 and 4,000 Hz may be the effect of healing after surgery. It suggests that clear indications of hearing preservation after direct FMT implantation against the RW without interposed fascia cannot be seen until 3 months after surgery. However, the results of BC thresholds over the 36-month follow-up confirm full hearing preservation after direct implantation of the FMT against the RW.
